# The implementation of teletherapy with patients with Severe Mental Illness during the COVID-19 first wave and its longitudinal association with hospitalisations: A retrospective multicenter study from Spain

**DOI:** 10.1192/j.eurpsy.2022.684

**Published:** 2022-09-01

**Authors:** A.J. Sánchez-Guarnido, B. Machado Urquiza, M.D.M. Soler-Sánchez, C. Masferrer, F. Perles, E. Petkari

**Affiliations:** 1 Hospital Santa Ana, Mental Health, Motril, Spain; 2 Hospital Psiquiátrico José Germain de Leganés, Mental Health, Madrid, Spain; 3 Centre Fòrum de l´Hospital del Mar., Institut De Neuropsiquiatria I Addiccions. Parc De Salut Mental, Barcelona, Spain; 4 Hospital de la Axarquía, Mental Health, Velez-Málaga, Spain; 5 Universidad Internacional de La Rioja, Health, Logroño, Spain

**Keywords:** teletherapy, severe mental illness (SMI), outpatient mental health, Covid-19

## Abstract

**Introduction:**

The COVID-19 related restrictions such as social distancing forced the search for feasible alternatives to the provision of care for patients with severe mental illness (SMI), with services opting for teletherapy as an substitute of face-to-face treatment.

**Objectives:**

To examine the implementation of teletherapy (telephone, videoconference) with patients with SMI during the COVID-19 first wave, and explore its associations with reduced hospitalisations after the first wave was over.

**Methods:**

We performed a retrospective assessment of 270 records of patients visiting fifteen outpatient mental health services across Spain during 2020. We retrieved sociodemographic and clinical data, including modality of received therapy (in-person, telephone, videoconference) in three time points (before, during and after the first COVID-19 wave) and hospitalisation rates two, four and six months later.

**Results:**

During the first wave, services implemented teletherapy (telephone and videoconference) extensively, whilst they reduced face-to-face therapy, though this returned to previous levels after the first wave. Hospitalisations two months later did not differ between patients who received teletherapy, and those who did not (*p*=.068). However, hospitalisations were lower for the first group of patients four (*p* =.004) and six months later (*p* <.001). Multilevel analyses suggested that receiving teletherapy by videoconference during the first wave was the factor that protected patients most against hospitalisations six months later (OR=0.25; *p*=.012).

**Conclusions:**

Our findings suggest that teletherapy plays a protective role against hospitalisations, especially when face-to-face therapy is not feasible. Therefore, it can be considered a valid alternative to ensure continuity of care to patients with SMI.

**Disclosure:**

No significant relationships.

